# The Bidirectional Relationship Between Sleep Disturbance and Functional Dyspepsia: A Systematic Review to Understand Mechanisms and Implications on Management

**DOI:** 10.7759/cureus.66098

**Published:** 2024-08-03

**Authors:** Alvin Billey, Asra Saleem, Bushra Zeeshan, Gayanthi Dissanayake, Meaza F Zergaw, Mohamed Elgendy, Sondos T Nassar

**Affiliations:** 1 Internal Medicine and Gastroenterology, California Institute of Behavioral Neurosciences and Psychology, Fairfield, USA; 2 Internal Medicine, California Institute of Behavioral Neurosciences and Psychology, Fairfield, USA; 3 Dermatology, California Institute of Behavioral Neurosciences and Psychology, Fairfield, USA; 4 Internal Medicine and Family Medicine, California Institute of Behavioral Neurosciences and Psychology, Fairfield, USA; 5 Medicine, California Institute of Behavioral Neurosciences and Psychology, Fairfield, USA; 6 Orthopaedics, California Institute of Behavioral Neurosciences and Psychology, Fairfield, USA; 7 Medicine and Surgery, California Institute of Behavioral Neurosciences and Psychology, Fairfield, USA

**Keywords:** functional dyspepsia, post prandial fullness, melatonin, circadian, epigastric pain, indigestion, disturbance, sleep, dyspepsia, functional

## Abstract

Functional dyspepsia (FD) is a prevalent chronic digestive disorder that significantly impacts patients' quality of life. Sleep disturbance (SD) is common among FD patients, yet the relationship between SD and FD remains poorly characterized. This systematic review explores the bidirectional relationship between FD and SD, investigating underlying mechanisms and implications for management. A rigorous and comprehensive systematic search was conducted across PubMed, PubMed Central (PMC), Google Scholar, Cochrane Library, and ScienceDirect using select keywords related to SD and FD. Only studies published in English from the past 10 years that met inclusion and exclusion criteria were included. Quality assessment tools specific to study types were employed to minimize bias. After applying inclusion and exclusion criteria and quality assessments, the review encompassed 30 studies. The key findings reveal that FD is frequently associated with SD, with a significant proportion of FD patients reporting poor sleep quality. The mechanisms linking SD and FD are complex, involving the circadian rhythm, visceral hypersensitivity, immune responses, and psychological factors. Nonpharmacological treatments like cognitive behavioral therapy (CBT), acupuncture, and pharmacological neuromodulators have shown promise in managing FD and SD, offering hope for improved patient outcomes. SD and FD share a significant bidirectional relationship, influenced by a complex interplay of physiological, psychological, and lifestyle factors. Addressing SD in FD patients may improve overall symptom management. Further research is crucial, as it should focus on isolating specific SD causes and their direct impacts on FD and other functional gastrointestinal disorders (FGIDs), opening up new avenues for understanding and treatment.

## Introduction and background

Indigestion, one of the most prevalent chronic digestive disorders, is a significant concern [1]. It may be due to an underlying condition that is detected or causes that cannot be detected, called functional dyspepsia (FD) [1]. FD is prevalent in approximately 10% of the United Kingdom and Canadian population, with a higher prevalence in the United States at approximately 12% [1]. This high prevalence underscores the urgent need for a deeper understanding and effective management of FD.

Rome IV criteria define FD as bothersome epigastric pain or burning at least once a day per week called epigastric pain syndrome (EPS), bothersome early satiety or postprandial fullness occurring at least three days per week called postprandial distress syndrome (PDS), or an overlap disorder termed EPS-PDS [2,3].

FD, a prevalent chronic digestive disorder, significantly impacts individuals' lives. It increases unnecessary healthcare usage, may cause somatoform-type behavior, and significantly impacts psychological well-being and quality of life. This includes higher rates of absenteeism from employment, lower productivity at work, reduced productivity at home, and increased medical and prescription medicine costs every year [4]. These implications underscore the need for a comprehensive approach to managing and treating FD.

FD frequently coexists with other functional gastrointestinal disorders (FGIDs), such as irritable bowel syndrome (IBS) and gastroesophageal reflux disease (GERD). These FGIDs may be associated concurrently with other non-gastrointestinal symptoms, including sleep disturbance (SD), anxiety, and depression, which can contribute to the maintenance or even progression of FGIDs [5].

While FD rarely leads to mortality, it significantly impacts the quality of life, with as many as 68% of patients reporting poor sleep quality [6]. There is evidence that points to a direct association between psychological distress and FD, although these studies are limited. However, SD, a common problem in FD patients, has a known association with and may influence the severity of dyspepsia symptoms [7].

Sleep is necessary for life and essential to physical and psychological health. The circadian rhythm regulates it. Over time, new lifestyles, such as shift work and overuse of electronic devices before bed, have adversely affected sleep [8]. Although SD is frequently found in patients with FGIDs, it is difficult to determine the cause and effect of the disturbances. However, we do know that SD worsens gastrointestinal symptoms, and conversely, many gastrointestinal diseases affect the sleep-wake cycle and lead to poor sleep [9].

Unlike GERD and IBS, the SD and FD relationship is still poorly characterized [10]. Although many studies have shown their association, how they contribute to symptom development or severity with each other has yet to be well documented. This study aims to provide insights into the bidirectional relationship between FD and SD and hopefully also provide evidence of the need for novel therapeutic approaches in persons suffering from both conditions.

## Review

This study is based on the Preferred Reporting Items for Systematic Reviews and Meta-Analyses (PRISMA) 2020 guidelines [11].

Eligibility criteria

This review question was formulated based on the participants, intervention, and outcome (PIO) elements: participants, patients diagnosed with FD or SD; intervention, investigating the bidirectional relationship between SD and dyspepsia, including exploring the impact of sleep quality on dyspepsia and the influence of dyspepsia on sleep patterns; and outcome, understanding the mechanisms underlying the bidirectional relationship between SD and dyspepsia such as alterations in neurotransmitter pathways, circadian rhythms, stress response, and gut-brain interactions. Additionally, exploring the implications of this relationship on managing dyspepsia and SD includes developing holistic management strategies targeting both conditions. Additional inclusion and exclusion criteria were added. Inclusion criteria were as follows: studies with human participants, English language (or include translations), and free full-text articles published within the past 10 years. In contrast, exclusion criteria were as follows: animal studies, papers published in languages other than English (without available translations), publications before 2014, and studies in persons without SD or FD.

Databases and search strategy

The search was conducted systematically using PubMed, PubMed Central (PMC), Google Scholar, Cochrane Library, and ScienceDirect databases. The last search of all the databases was performed on June 26, 2024. The key terms used in the search engines were sleep disturbance, functional dyspepsia, and the Medical Subject Heading (MeSH) strategy used in PubMed. Details of the databases and search strategies can be found in Table 1.

**Table 1:** Search strategy for different databases and their search result

Database	Keywords	Search strategy	No. of articles before filters	Filters	Search result
PubMed	Sleep, sleep disturbance, sleep quality, insomnia, dyspepsia, indigestion	Sleep disturbance OR Sleep OR Sleep Quality OR ((("Sleep"[Majr]) OR (“Sleep Wake Disorders/drug therapy"[Mesh] OR “Sleep Wake Disorders/metabolism"[Mesh] OR “Sleep Wake Disorders/microbiology"[Mesh])) OR "Sleep Quality"[Mesh]) OR "Sleep Initiation and Maintenance Disorders"[Mesh] AND Functional Dyspepsia OR Dyspepsia OR Indigestion OR "Dyspepsia"[Majr]) OR "Dyspepsia"[Mesh]	17002	Free full text, ten years, humans, English	1410
PubMed Central (PMC)	Sleep disturbance, functional dyspepsia	Sleep Disturbance AND Functional Dyspepsia	1536	Open access, ten years	718
Google Scholar	Sleep disturbance, functional dyspepsia	“Sleep Disturbance” AND “Functional Dyspepsia”	1320	English, ten years	872
Cochrane Library	Sleep disturbance, functional dyspepsia	Sleep AND “Dyspepsia”	200	English, ten years	102
ScienceDirect	Sleep disturbance, functional dyspepsia	Sleep Disturbance AND Functional Dyspepsia	1977	Open access, English, ten years	102

All references were grouped and organized using EndNote, and duplicate removal was done manually by EndNote. The records were manually screened based on the titles and abstracts, and irrelevant studies were excluded. The full-text articles for applicable studies were retrieved. All authors independently examined each article that was successfully retrieved according to the appropriate screening tool for quality appraisal to minimize the risk of bias in this study.

Risk of bias in individual studies

The full articles retrieved were assessed for quality assessment and risk of bias using tools depending on the study type: case-control and cohort studies, Newcastle-Ottawa Scale (NOS); cross-sectional studies, Appraisal tool for Cross-Sectional studies (AXIS) Critical Appraisal; narrative reviews, Scale for the Assessment of Narrative Review Articles 2 (SANRA 2); and systematic reviews and meta-analyses, Assessment of Multiple Systematic Reviews (AMSTAR 2). The assessment tools differed in their criteria and passing scores. For the paper to be accepted, a 70% score was required for each assessment tool. After completing the quality appraisal, 28 articles were included in this systematic review. Two authors agreed on which data to extract from the included articles.

Results

**Figure 1:**
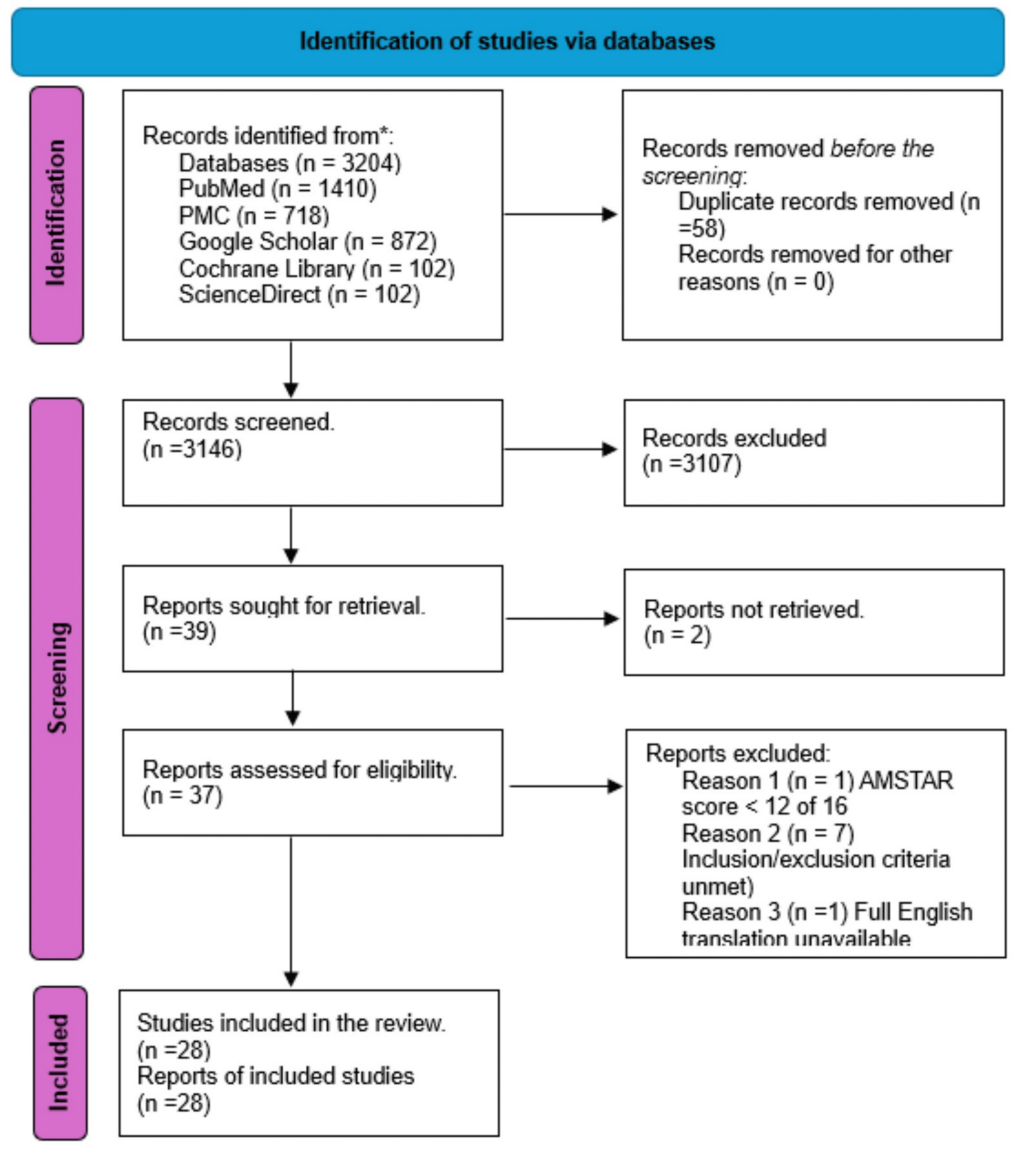
PRISMA 2020 flowchart of the databases and studies PRISMA: Preferred Reporting Items for Systematic Reviews and Meta-Analyses

This systematic review was carried out per the PRISMA criteria, which were used to screen and narrow down studies incorporated in this systematic review. It is outlined below in Figure 1.

Five databases were used to search for articles relevant to this study: PubMed, PMC, Google Scholar, Cochrane Library, and ScienceDirect. A thorough search was performed using the MeSH strategy outlined above and relevant regular keywords, which yielded 3204 results. From these, 41 studies were sought for retrieval, of which two were not retrieved; we subsequently applied our inclusion and exclusion criteria in addition to performing a quality appraisal for each study. This resulted in nine studies being eliminated, and ultimately, 28 studies were included in this systematic review. Table 2 below briefly describes the included studies regarding funding, population, setting, intervention, comparison, and outcomes.

**Table 2:** Description of articles included in this review IBS: irritable bowel syndrome; FD: functional dyspepsia; CBT: cognitive behavioral therapy; QOL: quality of life; SF-36: Short-Form 36-Item Health Survey

Author	Funding	Population	Intervention	Comparison	Setting	Outcome
Arnaout et al. [1]	Not specified	Adults in low- and middle-income countries	None (observational study)	None (cross-sectional study)	International, low- and middle-income countries	Prevalence and risk factors of FD
Barberio et al. [2]	Not specified	Patients diagnosed with Rome IV IBS and FD	None (observational study)	IBS alone vs. FD alone vs. overlap of both	Clinical setting, longitudinal follow-up	Natural history and clinical outcomes
Futagami et al. [3]	Yes	Patients with FD according to Rome IV criteria	None (observational study)	Subtypes of FD	Clinical setting	Classification and characteristics of FD subtypes
Barberio et al. [4]	Not specified	Global population with uninvestigated dyspepsia	None (meta-analysis)	Prevalence rates according to Rome criteria	Various studies included in the meta-analysis	Prevalence of uninvestigated dyspepsia
Colombo et al. [5]	Not specified	Children and adolescents with heartburn, FD, and/or IBS	None (observational study)	Presence vs. absence of sleep disturbances, anxiety, and depression	Clinical setting	Correlation of heartburn with sleep disturbances, anxiety, and depression
Huang et al. [6]	Not specified	Patients with FD	None (observational study)	Patients with sleep impairment vs. those without	Clinical setting	Correlation between sleep impairment and FD
Li et al. [7]	Not specified	Patients with FD diagnosed based on Rome III criteria	None (observational study)	Patients with sleep disturbances and psychological distress vs. those without	Clinical setting	Association between sleep disturbances, psychological distress, and FD
Li et al. [8]	Not specified	Patients experiencing sleep deprivation	Observation of sleep deprivation effects	NA	Clinical and experimental settings	Changes in gastrointestinal physiology and disease outcomes
Khanijow et al. [9]	Not specified	Patients with various gastrointestinal diseases	Observation of sleep dysfunction	Patients with and without sleep dysfunction	Clinical setting	Association between sleep dysfunction and gastrointestinal disease symptoms
Grover et al. [10]	Not specified	Patients with FD	Observation of sleep disturbances	Patients with and without sleep disturbances	Clinical setting	Correlation of sleep disturbances with FD symptoms
Alonso-Bermejo et al. [12]	Not specified	General population meeting Rome IV criteria for functional gastrointestinal disorders	Observation of prevalence	None	Community-based setting	Frequency of various functional gastrointestinal disorders
Zhao et al. [13]	Not specified	Patients with functional gastrointestinal disorders in class 3 hospitals in Tianjin, China	Observation of sleep quality	Patients with varying sleep quality	Hospital-based setting	Association between sleep quality and gastrointestinal disorder symptoms
Hyun et al. [14]	Not specified	General community with digestive symptoms	Observation of sleep disturbances	Individuals with and without sleep disturbances	Community-based setting	Correlation between digestive symptoms and sleep disturbances
Khan et al. [15]	Not specified	FD patients in a tertiary care hospital	Observation of sleep quality	Patients with varying sleep quality	Tertiary care hospital	Relationship between sleep quality and FD
Morito et al. [16]	Not specified	Individuals with abdominal symptoms	Observation of sleep disturbances	Individuals with and without sleep disturbances	Clinical setting	Correlation between sleep disturbances and abdominal symptoms
Su et al. [17]	Not specified	Patients with sleep disturbances	Observation of FD risk	Patients with and without sleep disturbances	Population-based cohort	Relative risk of FD
Topan and Scott [18]	Not specified	Individuals with disorders of gut-brain interaction	Observation of sleep patterns	Individuals with and without sleep disturbances	Clinical and research settings	Impact of sleep on gut-brain interaction disorders
Tseng and Wu [19]	Not specified	Rotating shift workers	Observation of circadian rhythm and sleep disturbance	Individuals with regular vs. rotating shifts	Occupational setting	Impact on gastrointestinal dysfunction
Du et al. [20]	Not specified	Patients with FD	Measurement of duodenal eosinophil degranulation	Patients with and without eosinophil degranulation	Clinical setting	Association between eosinophil degranulation and FD
Fowler et al. [21]	Not specified	Patients with disorders of gut-brain interaction	Observation of circadian rhythms and melatonin metabolism	Patients with normal vs. disrupted circadian rhythms	Clinical and research settings	Impact on gut-brain interaction disorders
Ermis et al. [22]	Not specified	Male patients with FD	Measurement of melatonin levels	Patients with varying melatonin levels	Clinical setting	Role of melatonin in FD pathogenesis
Fang et al. [23]	Not specified	Patients with FD	Observation of subgroups based on Rome III criteria	Different subgroups of FD	Clinical setting	Distinct aetiopathogenesis
Koloski et al. [24]	Not specified	Patients with IBS and FD	Observation of gut-brain and brain-gut pathways	Pathways in patients with IBS vs. FD	Population-based prospective studies	Independent pathways operating in IBS and FD
Nakamura et al. [25]	Yes	20 patients with FD and sleep disturbance	Administration of sleep aids for four weeks (zolpidem, eszopiclone, and suvorexant)	Pre- and post-intervention comparison of sleep quality and gastrointestinal symptoms	Clinical setting	Significant improvement in sleep quality and gastrointestinal symptoms. Improvement in QOL assessed by the SF-36. Significant reduction in anxiety scores
Law et al. [26]	Not specified	Patients with gastroduodenal disorders	CBT-based interventions	CBT interventions vs. other treatments	Various clinical trials included in the review	Effectiveness of CBT on gastroduodenal disorders
Ho et al. [27]	Not specified	Patients with FD	Acupuncture and related therapies	Acupuncture vs. prokinetics	Various studies included in the meta-analysis	Effectiveness of therapies on FD
Masuy et al. [28]	Not specified	Patients with FD	Various treatment options	Different treatments for FD	Review of clinical studies	Effectiveness and outcomes of treatments
Adibi et al. [29]	Yes	Individuals with and without FD	Observation of anxiety, depression, and psychological distress	FD patients vs. non-FD individuals	Community-based setting	Association between psychological factors and FD

Discussion

FD, a subset of FGIDs, is defined by the presence of dyspeptic symptoms, namely, epigastric pain or burning, and postprandial discomfort or early satiety, in a patient with no identifiable organic cause on an investigation, namely, an upper gastrointestinal endoscopy [12]. FGIDs include IBS, functional constipation (FC), GERD, and other symptoms associated with no structural or organic cause as classified by the Rome IV criteria for FGIDs. Compared to other FGIDs, FD patients have more trouble sleeping [13]. SD encompasses a variety of disorders that have all been documented in persons with a diagnosis of FD, including insomnia, hypersomnia, circadian rhythm sleep-wake disorders, sleep apnea, narcolepsy and cataplexy, parasomnia, and sleep-related movement disorders [14]. 

Digestive problems and sleep deprivation are frequently associated, but there is a complicated link between them. Dyspeptic symptoms, like abdominal pain, may make it difficult to fall asleep or postpone sleep onset, while insufficient sleep may worsen dyspepsia symptoms. A recent meta-analysis showed that individuals with postprandial distress (51%) and epigastric pains (40%) had a greater generalized incidence of sleep disruption [15]. Fass and colleagues found that 57.2% of patients studied with IBS and FD reported abdominal pain and discomfort that woke them from sleep. On the other hand, sleep deprivation has been reported to enhance gastrointestinal sensitivity and aggravate unpleasant abdominal symptoms [9,15]. This vicious cycle may explain the heightened symptoms of FD in patients with coexistent SD.

While the interrelation between SD and FD has been studied, its underlying mechanism is complex. Emerging insight into the pathophysiology of FD suggests that the circadian rhythm, visceral hypersensitivity, the immune system, and intestinal dysmotility are primary players in its pathogenesis [9,16]. The proposed underlying mechanisms emphasize the bidirectional system of our brain and gastrointestinal tract.

The circadian rhythm

Several brain regions regulate the sleep-wake state. The circadian rhythm originates in the suprachiasmatic nucleus (SCN) and regulates sleep-wake through hormones like melatonin and serotonin. The raphe nuclei are also involved in the circadian rhythm through their interaction with serotonin. The raphe nuclei receive signals from the gut's enteric nervous system via serotonergic, cholinergic, and noradrenergic pathways and, in turn, send signals to the dorsal column of the spinal cord that regulates gut motility and pain sensation. Serotonin, as a neurotransmitter, also enhances the activity of prokinetic neuropeptides in the gastrointestinal tract. Interference of these pathways through a sleep-wake cycle disturbance might affect gut motility. Overstimulating such pathways may cause hypersensitivity and bowel discomfort such as nausea, pain, or bloating [9,16,17].

Visceral hypersensitivity

Visceral hypersensitivity is a constant feature of FGIDs [18]. However, the mechanisms driving the response need to be better understood. There is a probable association with melatonin, which reduces pain symptoms of IBS by regulating the sleep-wake state. It's also plausible that poor sleep may affect the upper gastrointestinal tract, and the relationship between sleep and IBS may also apply to FD [17,18]. Night-time awakening disrupts sleep. This is frequently accompanied by emotional instability and psychosocial comorbidities that may induce visceral hypersensitivity, hyperalgesia, and hypervigilance, contributing to the symptoms in patients who suffer from FD [19].

The immune system

Eosinophil-mast cell-nerve interactions have an essential role in generating dyspeptic symptoms. Tryptase released from degranulated mast cells induces intestinal epithelial breakdown, activates other inflammatory cells, and raises visceral hypersensitivity in the intestinal tract. This low-grade mucosal inflammation, characterized by increased duodenal eosinophilia and mast cells in FD, may result from circadian disruption. However, this has mainly been unexplored so far [20,21]. Immune dysregulation causing FD could also be related to melatonin levels. Melatonin inhibits the acute neutrophilic response of inflammation. It maintains mucosal cell integrity by reducing neutrophil-mediated damage, suggesting alterations in melatonin release associated with irregular sleep patterns may contribute to inflammation [21,22]. However, it's important to note that the relationship between melatonin and the immune gastrointestinal response is much more complicated.

**Figure 2:**
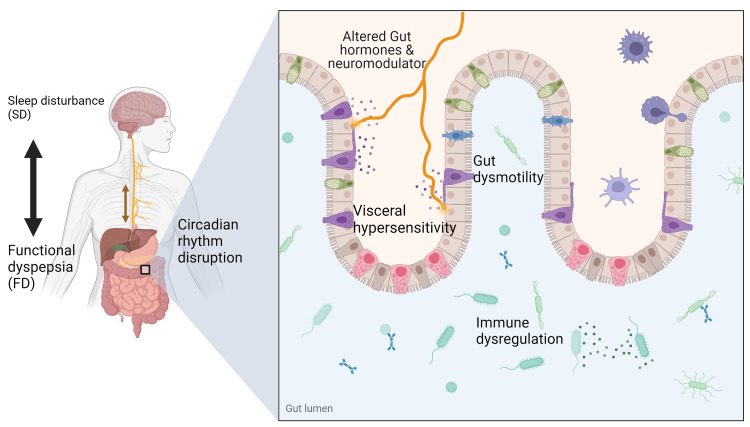
Gut-brain axis and its bidirectional communication Gut-brain interaction and bidirectional relationship of SD and FD. SD alters the circadian rhythm, affecting the release of neuromodulators and hormones such as melatonin, causing altered gut motility, visceral hypersensitivity, and immune dysregulation. Altered gut motility causing early satiety and abdominal discomfort is called PDS. Visceral hypersensitivity and immune dysregulation are associated with epigastric pain and are referred to as EPS. Sometimes with overlap (PDS-EPS). Symptoms of FD may cause waking or increased sleep latency, causing SD. Created with Biorender.com SD: sleep disturbance; FD: functional dyspepsia; PDS: postprandial distress syndrome; EPS: epigastric pain syndrome

Figure 2 below shows the bidirectional communication between the brain and the gastrointestinal tract, highlighting the main effects of SD on the circadian rhythm and gastrointestinal system.

Psychological and behavioral factors

**Figure 3:**
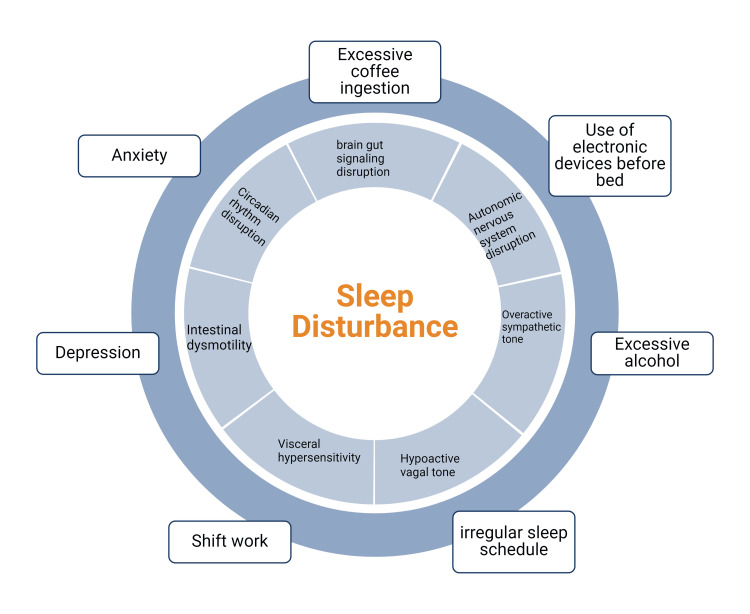
Lifestyle factors associated with SD and FD Common lifestyle factors affecting circadian rhythm and ultimately leading to SD. SD, in turn, has multiple effects on the gastrointestinal tract, which may cause or enhance symptoms of FD. Created with Biorender.com SD: sleep disturbance; FD: functional dyspepsia

Behavioral and lifestyle factors contributing to SD, such as coffee drinking, excessive alcohol intake, use of electronic devices before bed, anxiety, and depression, have also been linked with FD. This might be due to a disruption of brain-gut signaling and dysfunction of the autonomic nervous system, including an overactive sympathetic tone and a hypoactive vagal tone, which leads to visceral hypersensitivity and dyspeptic symptoms [23,24]. The autonomic dysfunction associated with depression might impair gastric accommodation and produce symptoms of FD, specifically the PDS subtype of the disease [23,24]. However, the gut and brain interact bidirectionally in FD. In people without FD, higher levels of anxiety and depression were significant predictors of developing IBS and FD. Also, between one-third and two-thirds of patients with FGIDs have a primary gut-driven syndrome that goes on to affect the brain [24]. Hypothesized pathways of gut-brain communication include direct secretion of neuroactive chemicals in the gut by bacteria, such as serotonin precursors, and stimulation of secretion of serotonin from enteroendocrine cells [24]. Figure 3 below highlights common lifestyle factors associated with SD and their effect on the gastrointestinal tract.

Therapeutic interventions, including nonpharmacological treatments

The connection between SD and FD continues to grow, with evidence showing that the use of sleep-inducing drugs is associated with reduced pain and overall improvement of dyspeptic symptoms in FD patients. So, it might be necessary to evaluate for SD in patients with FD and vice versa. If so, sleep aids may work for FD and SD [25]. There is a higher incidence of behavioral abnormalities and SD in patients with FD than those without FD. Cognitive behavioral therapy (CBT) is effective in patients with FD and SD. CBT-based interventions are associated with improvements in depression, anxiety, stress, health-related quality of life, and gastrointestinal symptoms [26]. Increased sensory signals from the gut and impaired central modulation of pain and gut functions are critical pathogenic features among FD patients. Acupuncture is a therapeutic approach suggested to modulate the brain-gut axis and restore homeostasis. This potential mechanism might explain the effectiveness of acupuncture for managing FD [27]. As the name implies, the brain‐gut axis is a bidirectional communication pathway between the brain and the gut, connecting the central and enteric nervous systems. This pathway is frequently disturbed in patients who experience SD. Neuromodulators, like antidepressants and anxiolytics, are second-line drugs indicated for refractory FD symptoms through their pain‐modulating potential on the brain-gut axis. Psychiatric comorbidities that are associated with SD, such as depression or anxiety disorders, frequently coexist with FD, for which neuromodulators are used. Although several have been studied, tricyclic antidepressants (TCAs) are the most effective [28].

SD and FD share a bidirectional relationship. However, other factors also play a role, and the current study has limitations. Psychological factors, such as stress, anxiety, and depression, affect both conditions [29]. More research is needed to assess the specific causes of SD and its relationship with FD. Rome IV criteria increased the sensitivity for the diagnosis of FD by introducing a third category of FD with an overlap of symptoms. This new category, PDS-EPS overlapped syndrome, increased the sensitivity of FD detection [3]. Our data set included research dating back to 2014, before the introduction of Rome IV, which may have caused some heterogeneity in how FGIDs are classified among studies included in this review.

## Conclusions

The systematic review highlights a significant bidirectional relationship between SD and FD. There is evidence suggesting that individuals with SD are more likely to experience FD and, conversely, those with FD are more likely to suffer from SD. A complex interplay of physiological, psychological, and lifestyle factors influences this relationship. SD affects the circadian rhythm, alters visceral sensitivity, and affects the immune system. Nonpharmacological treatment modalities such as CBT, acupuncture, and pharmacological neuromodulators are effective at improving symptoms of SD and FD and may be the best option for patients with both conditions. More research is needed to isolate the relationship between specific causes of SD, such as insomnia, hypersomnia, circadian rhythm sleep-wake disorders, sleep apnea, narcolepsy and cataplexy, parasomnia, sleep-related movement disorders, and their association with other FGIDs.
